# CAN Optical Coherence Tomography redefine amblyopia?

**DOI:** 10.22336/rjo.2017.18

**Published:** 2017

**Authors:** Elena Avram

**Affiliations:** * Ophthalmology Department, Medlife Băneasa Hyperclinic, Bucharest, Romania

**Keywords:** amblyopia, Optical Coherence Tomography, macular thickness, choroid thickness

## Abstract

**Introduction:** For many years, amblyopia was regarded as a disorder of the visual system in which an organic cause could not be identified. Optical Coherence Tomography opens new horizons in understanding the etiopathology of amblyopia and seems to highlight morphologic anomalies in the retina of the amblyopic eye.

**Purpose:** The objective of this paper is to analyze the macular thickness, optic nerve changes, and choroidal thickness found in patients diagnosed with amblyopia based on trials reported in the literature.

**Material and methods:** This study analyzes 30 clinical trials regarding amblyopia evaluation with Optical Coherence Tomography. The research articles analyzed were published between 2006 - 2016 and were identified on PubMed database.

**Results:** 19 research studies focused on macular and nerve optic changes, 7 on choroidal changes and 6 on retinal changes after occlusion. The results were discussed according to the type of amblyopia, alteration of macular thickness, optic nerve changes, ganglion cell layer changes, and alteration of choroidal thickness.

**Conclusions:** The results are of great variability, and it seems that macula and choroid involvement is more frequently suggested compared with optic nerve involvement.

**Abbreviations:** OCT = Optical Coherence Tomography, RNFL = Retinal Nerve Fiber Layer, GCC = Ganglion Cell Complex, ACD = Anterior Chamber Depth, BCVA = Best Corrected Visual Acuity

## Introduction

For many years, amblyopia has been considered a disorder of the visual system that represents unilateral or bilateral reduction of visual acuity in which an organic cause could not be detected [**1**].

New horizons in understanding the etiopathology of amblyopia are offered by Optical Coherence Tomography (OCT) which seems to highlight morphologic anomalies in the retina of the amblyopic eye. 

A series of studies that aimed to analyze macular thickness, optic nerve morphology and also choroidal thickness in the amblyopic eye have been published in the last years. The results are often contradictory because, as Kim says, when conducting an OCT in patients with anisometropia it is important to correct the magnification of the device according to refraction and axial length of the eye. Also, results differ depending on the device used [**2**].

Another limitation regarding correct interpretation of OCT in children emerges from the inexistence of international established normative values of macular and retinal nerve fiber layer (RNFL) parameters in children [**3**].

## Material and methods 

30 clinical trials published between 2006 and 2016 regarding OCT evaluation in amblyopia were identified on PubMed database and results were discussed according to:

- Type of amblyopia;

- Alteration of macular thickness;

- Optic nerve and ganglion cell layer changes;

- Alteration of choroidal thickness;

- Retinal morphologic changes after occlusion.

## Results

• **Macular thickness (volume)**

Using OCT equipment, several researchers analyzed in their studies morphologic changes in the macula thickness of amblyopic patients and the results are of great variability (**Table 1**).

**Table 1:** Macular thickness in amblyopic eye [**2**,**4**-**7**,**10**,**12**,**17**-**19**,**32**]

	Patient age	Type of amblyopia		**Mean central macular thickness** (µm)		
			*Amblyopic eye*	*Contralateral eye*	*Normal subject eye*	*P*
Alotaibi (2011)	5 – 12 years	Anisometropia, Strabismus	259.3 ± 16.67	255.6 ± 21.34	–	0.195
Al-Haddad (2011)	20 ± 12 years	Anisometropia, Strabismus	273.8	–	257.9	0.001
Wang (2012)	7 – 11 years (8.82 ± 1.47)	Anisometropia	157.96 ± 15.82	151.72 ± 13.95	–	0.045
Firat (2013)	5 – 23 years (12.6 ± 5.4)	Anisometropia, Strabismus	258.25 ± 18.31	258.75 ± 19.54	248.62 ± 10.57	0.06
Wu (2013)	5 – 16 years (9.7 ± 1.9)	Anisometropia	257.1 ± 15.8	258.6 ± 13.9	–	0.80
Kim (2013)	7.45 ± 2.57 years	Congenital cataract	237.05 ± 37.74	226.67 ± 34.71	233.74 ± 27.11	0.137 0.792
Araki (2014)	4 – 18 years (8.5 ± 3.5)	Anisometropia	236.90 ± 18.11	231.67 ± 15.17	–	0.099
Yalcin (2014)	8 – 14 years (10.5)	Anisometropia	220 ± 38.25	202.87 ± 31.01	198.91 ± 22.50	0.025
Yakar (2015)	18 – 55 years (34.7 ± 11.83)	Anisometropia	266.90 ± 23.22	263.90 ± 22.84	–	0.342
Demircan (2015)	5 – 12 years	Anisometropia	260.71 ± 14.48	254.29 ± 14.79	–	0.001
	13 – 42 years		265.61 ± 22.42	267.11 ± 24.52	–	0.483

Regarding anisometropic amblyopia:

- Park found in the group of patients he studied in 2011 that the ganglion cell layer thickness and internal plexiform layer were thinner than in the control group. He also noticed that other retinal layers (nerve fibre layer, inner nuclear layer, outer plexiform layer, outer nuclear layer) presented significant differences in thickness at several macular locations [**8**];

- Wang (2012) stated that the average thickness of the foveola is thicker than in normal eyes, but other regions have no significant difference [**4**];

- Wu (2013) showed in his study that hyperopic anisometropic amblyopic eyes have a thicker foveola than the contralateral eyes [**5**];

- Araki (2014) concluded that there are significant differences between amblyopic and fellow eyes that are independent of abnormalities in the visual cortex. He showed that the macular outer retinal thickness (1 and 3 mm regions) is significantly thicker in „lazy eyes” [**6**];

- Demircan (2015) discovered that in patients age 5-42 years the mean central macular thickness in the amblyopic eyes was thicker patients under 12 years [**7**].

After studying anisometropic and strabic ambliopic patients:

- The Sydney Childhood Eye Study (2009) affirmed that in children aged 6 to 12 years, central macular thickness may be increased in „lazy eyes”, although it is uncertain if this precedes or follows the development of amblyopia [**9**];

- Al-Haddad (2011) showed in his study that the mean macular thickness was significantly increased in amblyopic eyes compared with fellow eyes. This difference remained significant in the anisometropic group but not in the strabic group. The mean RNFL thickness was similar in amblyopic and fellow eyes [**10**]; 

- In 2013 Tugcu claimed that in patients with combined (anisometropic and strabismic) amblyopia and in patients with anisometropic amblyopia the thickness of ganglion cell complex (GCC) was significantly increased in both amblyopic and nonamblyopic eyes compared to healthy patients. The strabismic amblyopia group presented with significant reduction in GCC thickness and increased foveal thickness compared to nonamblyopic eyes. He did not found significant differences in morphological and functional measures among amblyopic groups but he detected significant differences in the retinal morphology of both amblyopic and nonamblyopic eyes compared with healthy control subjects [**11**];

- Rajavi (2014) showed that the average foveal thickness of children with moderate and severe amblyopia was 10 microns thicker compared with the congener eye or the control group. Also, male patients had a mean macular thickness thicker than female patients and there was no correlation between the types of amblyopia and macular morphology changes [**12**];

- Szigeti (2014) revealed that there are subtle changes that affect the outer nuclear layer of the fovea suggesting the possible involvement of the photoreceptors [**13**]. 

Concerning deprivational amblyopia:

- Kim (2013) investigated OCT changes in deprivational amblyopia due to congenital cataract and concluded that the mean macular thickness in amblyopic eyes was not different from the contralateral eye or control group, but noticed a thickening of the nasal RNFL compared with the fellow eye or control group [**2**]; 

- Long (2016) stated that 3 months after surgery, unilateral pediatric cataract patients show no significant interocular anatomical parameters differences except for anterior chamber depht (ACD) and that the parameters evaluated by OCT were not significantly correlated with the final BCVA [**14**]. 

Also, there are scientists like Kee (2006) Repka (2009), Firat (2013) and Yakar (2015) who claim the inexistence of differences between amblyopic patients and the control group in their studies regarding macula morphology [**15**-**18**].

• **RNFL and Optic nerve changes**

Studies regarding morphologic changes of the RNFL and optic nerve disc in amblyopic patients highlighted with the help of OCT show inconstancy in finding a pattern (**Table 2**).

Wu (2013) affirmed in his study that hyperopic anisometropic amblyopic eyes have thicker peripapillary RNFL than the contralateral eyes [**5**]. Also, after analyzing patients with anisometropic amblyopia Araki (2014) concluded that there are significant differences between amblyopic and fellow eyes: mean RNFL thickness significantly thicker, rim area significantly larger and smaller C/D ratio, in the amblyopic eyes [**6**].

Some scientist such as Kee (2006), Repka (2009), Huynh (2009), Firat (2013), Yalcin (2014), Demircan (2015) and Yakar (2015) claimed that there are no differences in RNFL and optic nerve disc morphology between amblyopic patients and control groups [**7**,**9**,**15**-**19**].

**Table 2:** RNFL Thickness in amblyopic eye [**2**,**5**-**7**,**12**,**15**-**19**,**32**]

	Patient age	Type of amblyopia		**RNFL Thickness** (µm)		
			*Amblyopic eye*	*Contralateral eye*	*Normal subject eye*	*P*
Kee (2006)	4 – 17 years (8.5)	Anisometropia, Strabismus	107.2 ± 16.2	106.7 ± 16.5	108.8 ± 11.3	0.810 0.615
Repka (2009)	9.2 ± 1.5 years	Anisometropia, Strabismus	111.4 (10.4)	109.6 (11.4)	–	0.13
Alotaibi (2011)	5 – 12 years	Anisometropia, Strabismus	112.16 ± 12.67	106 ± 8.91	–	<0.0001
Wu (2013)	5 – 16 years (9.7 ±1.9)	Anisometropia	113.9 ± 7.2	109.2 ± 6.9	–	0.02
Firat (2013)	5 – 23 years (12.6 ± 5.4)	Anisometropia, Strabismus	113.22 ± 21.47	111.57 ± 18.25	109.96 ± 11.31	0.13
Kim (2013)	7.45 ± 2.57 years	Congenital cataract	99.64 ± 10.11	97.28 ± 12.34	95.38 ± 9.74	0.429 0.286
Araki (2014)	4 – 18 years (8.5 ± 3.5)	Anisometropia	112.5 ± 9.21	107.03 ± 8.74	–	0.004
Yalcin (2014)	8 – 14 years (10.5)	Anisometropia	101 ± 10.77	104.4 ± 10.95	105.08 ± 10.10	0.285
Yakar (2015)	18 – 55 years (34.7 ± 11.83)	Anisometropia	111.90 ± 12.94	109.70 ± 9.42	–	0.621
Demircan (2015)	5 – 12 years	Anisometropia	111 ± 19.23	105.67 ± 14.77	–	0.848
	13 – 42 years		103.69 ± 8.3	100.92 ± 7.61	–	0.363

**• Choroidal thickness**

Morphological changes of the choroid evidenced with the help of OCT were studied by some researchers who certified that in amblyopic patients the subfoveolar area is significantly higher than in the congener eye or control group [**20**-**25**]. However, Celik (2016) stated that, in young Turkish adults with hyperopic anisometropic amblyopia, the subfoveal choroidal, ganglion cell complex, macular and RNFL thickness measurements were not statistically significant between hyperopic anisometropic amblyopic eyes and normal fellow eyes [**25**].

**• Are there morphologic changes after occlusion?**

6 studies were identified regarding the effect of optical correction and occlusion on the retina, optic nerve and choroid morphology. Oner (2016) analyzed central choroidal thickness before and after 6 months of occlusive treatment and reported no thickness reduction of the central choroid after amblyopia treatment. While Miki (2010), Tugcu (2013), and Yassin (2015) did not found differences between macular thickness and RNFL thickness before and after occlusion therapy, Pang (2011) stated that the central macular thickness was reduced after treatment compared with peripheric macular thickness in which changes were not noticed [**26**-**30**]. 

Also, Liu (2010) stated that some children with various types of amblyopia who failed to achieve normal visual acuity after treatment show macular abnormality on OCT examination [**31**].

## Conclusions 

Today, with the help of OCT, a new controversy regarding the presence of morphologic retinal changes in patients diagnosed with amblyopia arises. This opens new horizons in better defining amblyopia and can confirm if ocular structure are affected or not. In the last years, scientists have been trying to determine if morphologic changes in the macula, optic nerve, or choroid of amblyopic patients exist. The results are of great variability and, it seems that macula and choroid involvement is more frequently suggested compared with optic nerve involvement.

**Disclosure**

None.
